# Pseudo-Hyperaldosteronism After the Ingestion of Licorice Tea: A Case Report

**DOI:** 10.7759/cureus.109726

**Published:** 2026-05-27

**Authors:** Clara Guarneri, Léticia Al-Kai

**Affiliations:** 1 Emergency Department, Hôpital Delta, Chirec, Bruxelles, BEL

**Keywords:** hypertension, hypokalemia, licorice, pseudo-hyperaldosteronism, renin-aldosterone

## Abstract

Severe hypokalemia is a common electrolyte disorder with a broad spectrum of etiologies. We report the case of a 44-year-old woman admitted to the emergency department with nausea, vomiting, chills, and chronic diffuse paresthesia. On admission, the patient was hypertensive. Laboratory evaluation revealed profound hypokalemia (2.3 mmol/L), severe hypophosphatemia, metabolic alkalosis, and electrocardiographic abnormalities, including U waves and a prolonged corrected QT interval, a key marker of severity associated with a high risk of life-threatening arrhythmias. Further investigations demonstrated inappropriate renal potassium wasting in the setting of suppressed plasma renin and aldosterone levels.

A detailed dietary history uncovered the excessive consumption of licorice tea, approximately 2 L daily over a year, leading to the diagnosis of licorice-induced pseudo-hyperaldosteronism. In this context, associated electrolyte disturbances, including hypophosphatemia and hypomagnesemia, are likely secondary to mineralocorticoid receptor activation and renal tubular losses rather than a direct effect of licorice.

The patient required admission to the intensive care unit for aggressive potassium repletion and antihypertensive therapy. Following cessation of licorice intake, electrolyte abnormalities and blood pressure gradually normalized.

This case highlights the critical importance of obtaining a thorough dietary history in patients with unexplained severe hypokalemia. Although uncommon, licorice intoxication represents a reversible cause that can be rapidly corrected with appropriate recognition and management. It also underscores the prognostic significance of QTc prolongation in severe electrolyte disturbances and the need for prompt cardiac monitoring.

## Introduction

Hypokalemia is a frequently encountered electrolyte abnormality in both emergency and internal medicine, with multiple potential causes including gastrointestinal losses, renal disorders, and endocrine disturbances. It is one of the most common electrolyte disturbances seen in clinical practice and may lead to severe and potentially life-threatening complications if not promptly recognized, including electrocardiographic abnormalities, QT prolongation, as observed in our patient (QTc 604 ms), and ventricular arrhythmias [1,2].

Licorice-induced pseudo-hyperaldosteronism is a rare but well-documented condition resulting from the excessive consumption of glycyrrhizin-containing products. The active metabolites of glycyrrhizin inhibit 11β-hydroxysteroid dehydrogenase type 2 (11β-HSD2), leading to apparent mineralocorticoid excess, which can manifest as severe hypokalemia, hypertension, and metabolic alkalosis [3,4]. To date, no robust epidemiological data are available regarding its prevalence or incidence. The existing literature mainly consists of isolated case reports and small case series, suggesting that this condition is rare and likely underdiagnosed. The cases that have been reported in the literature are most commonly associated with excessive intake of herbal teas, candies, traditional remedies, chewing tobacco, or over-the-counter supplements containing licorice extract. 

We report a case illustrating this reversible yet underrecognized cause of profound electrolyte disturbance, highlighting the importance of recognizing dietary and herbal contributors in patients presenting with severe hypokalemia, as well as the potential for life-threatening cardiac complications.

## Case presentation

A 44-year-old woman presented to the emergency department with nausea, vomiting, and chills that had begun that same day along with paresthesia, primarily around the mouth, that had been present for a year. She had no significant past medical history, has never used drugs, and was not taking any prescribed medications. She had seen her general practitioner a few weeks earlier because of paresthesia. He had prescribed a multivitamin regimen. 

Physical examination was initially unremarkable, with no focal neurological deficits and a normal cardiopulmonary examination.

Her vital signs on admission were as follows: blood pressure 170/90 mmHg, heart rate 83/min, temperature 36.9°C, oxygen saturation 100%, and BMI 19.5 kg/m^2^.

Initial laboratory investigations revealed severe hypokalemia (2.3 mmol/L) and severe hypophosphatemia (Table 1).

**Table 1:** Laboratory results at hospital admission MCV: mean corpuscular volume; PT: prothrombin time; INR: international normalized ratio; APTT: activated partial thromboplastin time; ASAT: aspartate aminotransferase; ALAT: alanine aminotransferase; GGT: gamma-glutamyl transferase; PAL: alkaline phosphatase; LDH: lactate dehydrogenase; CRP: C-reactive protein; TSH: thyroid-stimulating hormone; T4: thyroxine; T3: triiodothyronine

Markers	Values	Units	Normal values
Red blood cells	4.301	10^6^/mm^3^	3.8-5.1
Hemoglobin	14.1	g/dL	11.7-15.5
Hematocrit	38.9	%	35-45
MCV	90	fL	81-100
Platelets	188	1000/mm^3^	150-400
White blood cells	7.4	1000/mm^3^	4.5-11
Neutrophils	6.36	1000/mm^3^	1.8-7.7
Eosinophils	0.03	1000/mm^3^	0-0.45
Basophils	0.02	1000/mm^3^	0-0.2
Lymphocytes	0.6	1000/mm^3^	1-4.8
PT	77	%	>70
INR	1.2	-	<1.5
APTT	25.9	seconds	25-37
Fibrinogen	243	mg/dL	200-393
Sodium	141	mmol/L	136-145
Potassium	2.3	mmol/L	3.5-5.1
Chloride	96	mmol/L	98-107
Phosphate	0.27	mmol/L	0.81-1.45
Calcium	2.18	mmol/L	2.15-2.5
Bicarbonate	23	mmol/L	22-29
Magnesium	0.69	mmol/L	0.66-0.9
Urea	25	mg/dL	15-40
Creatinine	0.67	mg/dL	0.5-0.9
Total bilirubin	1.1	mg/dL	<1.2
Direct bilirubin	0.4	mg/dL	<0.3
ASAT	33	U/L	<32
ALAT	27	U/L	<33
GGT	8	U/L	<36
PAL	45	U/L	35-104
LDH	217	U/L	135-214
CRP	4	mg/L	<5
Glucose	118	mg/dL	74-109
TSH	2.04	mU/L	0.27-4.2
T4	13.1	pmol/L	12-22
T3	3.29	pmol/L	3.1-6.8
Vitamin D	18	ng/mL	>30
Parathormone	168	ng/L	17.3-84.6

Arterial blood gas analysis revealed metabolic alkalosis (Table 2).

**Table 2:** Arterial blood gas analysis with FiO₂ 21% and temperature 37°C pCO2: partial pressure of carbon dioxide; pO2: partial pressure of oxygen; FiO₂: fraction of inspired oxygen

Markers	Values	Units	Normal values
pH	7.59		7.35-7.45
pCO2	32	mmHg	35-45
pO2	79	mmHg	83-108
Ionized calcium	0.87	mmol/L	0.36-0.75
Lactate	0.8	mmol/L	1.15-1.33

**Figure 1:**
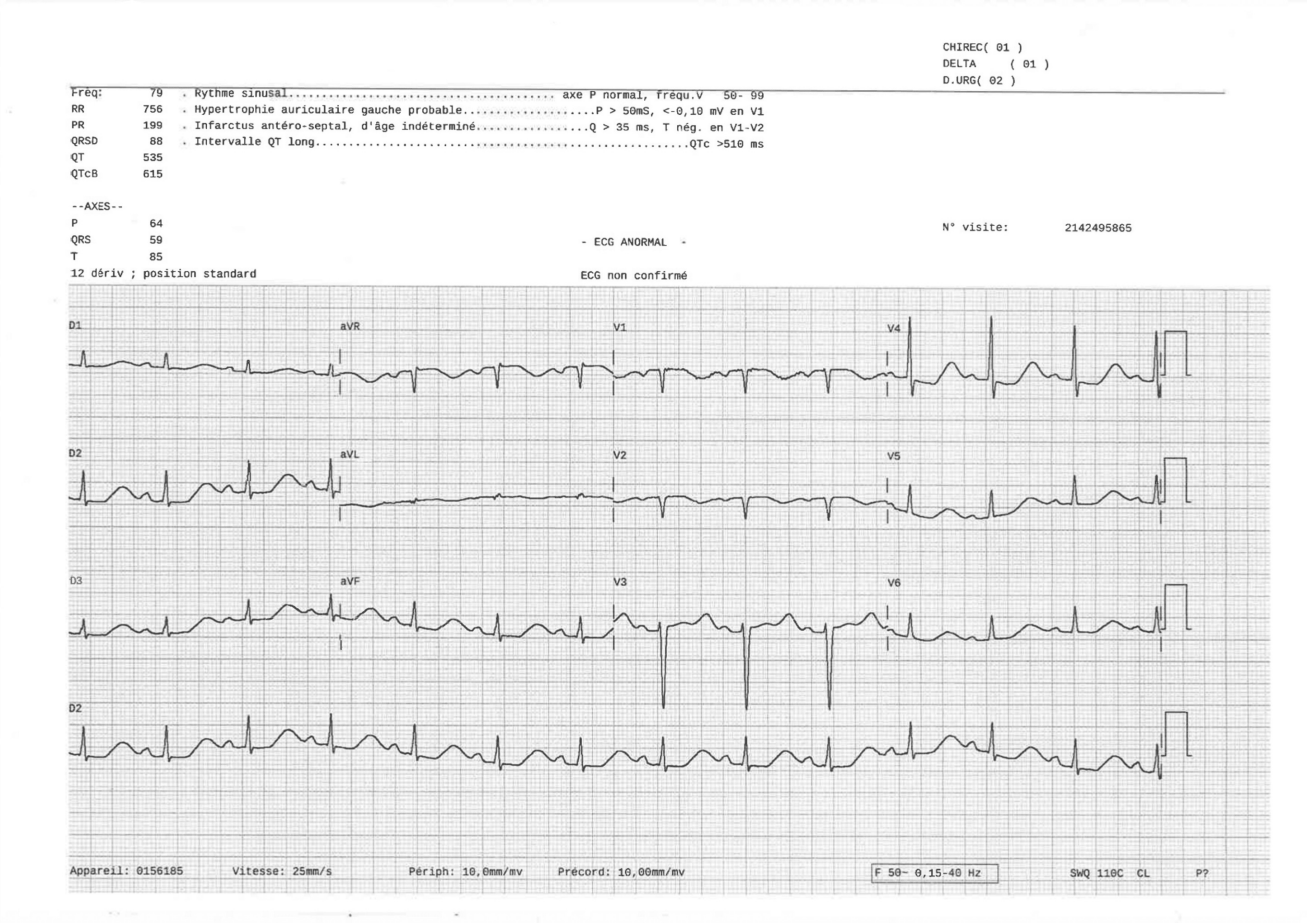
ECG showing U waves and a prolonged corrected QT interval (604 ms) ECG: electrocardiogram

An electrocardiogram (ECG) was performed and showed U waves and a prolonged corrected QT interval (604 ms), raising concern for potentially life-threatening arrhythmias (Figure 1). 

A detailed dietary history revealed the excessive consumption of licorice tea, approximately 2 L daily for several weeks. She drank licorice tea purely for pleasure because she liked the taste, and she does not mention any other reasons for her consumption. She did not consume licorice in any other form. She also reported that approximately three years earlier, she had lost 8 kg through diet and exercise. However, her weight had remained stable since then, and she maintained a varied diet without any restrictions.

Urinary studies demonstrated inappropriate renal potassium wasting, and endocrine evaluation showed markedly suppressed renin and aldosterone levels (Table 3, Table 4).

**Table 3:** Additional laboratory findings

Markers	Values	Units	Normal values
Renin	<0.5	mcg/mL	4.4-46.3
Aldosterone	16.8	pg/mL	17.6-232

**Table 4:** Biochemical analysis of a spot urine sample There are no reference values as this is simply a spot urine sample. Reference values apply only when the analysis is based on a 24-hour urine sample.

Markers	Values
Creatinine	27 mg/dL
Sodium	109 mmol/L
Potassium	51.3 mmol/L
Chloride	91 mmol/L
Urea	585 mg/dL
Glucose	858 mg/dL

Based on the clinical, laboratory, and hormonal findings, a diagnosis of licorice-induced pseudo-hyperaldosteronism was established.

Management required admission to the intensive care unit for continued monitoring, aggressive intravenous potassium replacement (total of 300 mEq over 24 hours), and the initiation of antihypertensive therapy. Spironolactone 25 mg once a day was started, followed by bisoprolol 2.5 mg twice a day, leading to progressive correction of electrolyte disturbances and improved blood pressure control. No arrhythmias were detected during monitoring in the intensive care unit. The hypokalemia gradually resolved, and the QTc interval returned to normal. After clinical stabilization, the patient was transferred to the nephrology ward and subsequently discharged with adjusted oral medications and strict instructions to discontinue licorice consumption.

At the one-month follow-up, serum electrolyte levels had normalized, and blood pressure was within the low-normal range, allowing the discontinuation of antihypertensive therapy. This case highlights the importance of recognizing excessive licorice intake as a reversible cause of severe hypokalemia and pseudo-hyperaldosteronism.

## Discussion

From a pathophysiological perspective, licorice contains glycyrrhizin, which is metabolized into glycyrrhetinic acid. This metabolite inhibits 11β-HSD2, an enzyme expressed in renal collecting duct cells. Under physiological conditions, 11β-HSD2 converts active cortisol into its inactive form, cortisone, thereby preventing the inappropriate activation of mineralocorticoid receptors by cortisol [3]. 

Excessive licorice consumption leads to the inhibition of 11β-HSD2 [5]. As a result, cortisol, present at plasma concentrations significantly higher than aldosterone, binds to and activates mineralocorticoid receptors, inducing a state of apparent mineralocorticoid excess, also referred to as pseudo-hyperaldosteronism. This condition promotes sodium retention and renal potassium wasting, leading to hypokalemia, arterial hypertension, and occasionally metabolic alkalosis. As for the other electrolyte disturbances, they are explained by the same underlying pathophysiological mechanism. Regarding hypomagnesemia, it primarily results from renal magnesium wasting secondary to the activation of the mineralocorticoid receptor, further exacerbated by the associated hypokalemia, which promotes urinary magnesium loss and perpetuates a vicious electrolyte cycle.

Although the clinical presentation resembles secondary hyperaldosteronism, serum aldosterone levels are typically suppressed due to the inhibition of the renin-angiotensin-aldosterone system secondary to sodium overload.

Clinically, our patient presented with limb paresthesias. Laboratory evaluation revealed severe hypokalemia, suppressed aldosterone levels, and increased urinary potassium excretion, in association with arterial hypertension. In published reports, the most common clinical manifestations include hypertension and hypokalemia. The paresthesias observed in our patient are likely explained by neuromuscular hyperexcitability induced by metabolic alkalosis associated with pseudo-hyperaldosteronism. Alkalosis promotes a reduction in circulating ionized calcium, a well-known mechanism responsible for perioral paresthesias. The associated electrolyte abnormalities, particularly hypokalemia and hypomagnesemia, likely further contributed to increased neuronal excitability. Neuromuscular symptoms such as fatigue, fasciculations, or clonus may also occur and, in severe cases, can progress to rhabdomyolysis [2]. As for the hypophosphatemia observed in our patient, it was caused by vomiting and respiratory alkalosis. In fact, hypophosphatemia is not typically seen in hyperaldosteronism*.*

Severe hypokalemia is strongly associated with ventricular repolarization abnormalities, including QTc prolongation, which may predispose to early afterdepolarizations and life-threatening ventricular arrhythmias such as torsades de pointes. Although no strict threshold exists, the risk of malignant arrhythmias markedly increases when serum potassium falls below 3.0 mmol/L and becomes particularly significant below 2.5 mmol/L, especially in the presence of additional risk factors. These findings highlight the need for prompt correction and close cardiac monitoring in patients presenting with severe hypokalemia [6].

Diagnosis relies primarily on a thorough medical history in the appropriate clinical context suggestive of hyperaldosteronism. A meta-analysis demonstrated that chronic oral intake of glycyrrhizin exceeding 100 mg/day is significantly associated with an increased risk of hypertension and hypokalemia [7]. However, excessive licorice consumption does not invariably result in clinical or biochemical abnormalities. Toxicity depends on multiple factors, including the ingested dose, the type of licorice-containing product, acute versus chronic exposure, and interindividual susceptibility [8]. Both the duration of exposure and the daily amount consumed are relevant determinants of toxicity [3]. Intake exceeding 30 days has been shown to be statistically associated with adverse effects, consistent with our patient's history of licorice consumption over several months [3].

Management primarily consists of the discontinuation of licorice intake, often combined with potassium supplementation. Normalization of clinical and laboratory parameters may require several days to several weeks. In our case, the patient received up to 300 mEq/day of intravenous potassium chloride, in addition to three daily oral doses of potassium supplementation (Ultra-K, 30 mEq per dose). Antihypertensive therapy with spironolactone 50 mg and bisoprolol 5 mg was also initiated. Mineralocorticoid receptor antagonists such as spironolactone are the antihypertensive drugs of choice because they block aldosterone receptors in the kidney. This leads to reduced sodium reabsorption, reduced fluid retention, and reduced potassium excretion [9].

## Conclusions

This finding highlights licorice-induced pseudo-hyperaldosteronism as a rare, reversible, and often overlooked cause of severe hypokalemia. It underscores the critical importance of a detailed medical history, particularly regarding drugs, diet, and herbal remedies, in any patient presenting with unexplained severe hypokalemia. Furthermore, it should be noted that herbal products, such as herbal teas containing licorice, are not without risks and may be responsible for potentially serious endocrine and cardiovascular complications.

In addition, severe hypokalemia may lead to life-threatening cardiac arrhythmias, including torsades de pointes, particularly in the presence of marked QT prolongation. Therefore, prompt electrocardiographic evaluation and continuous cardiac monitoring are essential until complete correction of electrolyte abnormalities.

Early identification of this etiology allows for simple and effective management based on discontinuing licorice consumption, thereby avoiding unnecessary diagnostic investigations.

Given the rarity and variable clinical presentation of licorice-induced pseudo-hyperaldosteronism, clinical assessment should be individualized according to each patient's exposure history, biochemical profile, and severity of presentation.
